# Evolutionary trade-offs between intergenerational and transgenerational fitness effects

**DOI:** 10.1093/evlett/qrag031

**Published:** 2026-07-30

**Authors:** Isaac Harris, Elizabeth M L Duxbury, Tracey Chapman, Simone Immler, Alexei A Maklakov

**Affiliations:** School of Biological Sciences, University of East Anglia, Norwich Research Park, Norwich, United Kingdom; School of Biological Sciences, University of East Anglia, Norwich Research Park, Norwich, United Kingdom; Department of Zoology, University of Cambridge, Cambridge, United Kingdom; School of Biological Sciences, University of East Anglia, Norwich Research Park, Norwich, United Kingdom; School of Biological Sciences, University of East Anglia, Norwich Research Park, Norwich, United Kingdom; School of Biological Sciences, University of East Anglia, Norwich Research Park, Norwich, United Kingdom

**Keywords:** adaptation, evolution, intergenerational epigenetic inheritance, transgenerational epigenetic inheritance, nongenetic inheritance, transgenerational trade-offs

## Abstract

Intergenerational and transgenerational fitness effects can shape evolutionary processes. Theoretically, however, intergenerational and transgenerational effects can trade off with each other with profound consequences for evolutionary processes. Here, we show that beneficial intergenerational effects that increase offspring fitness can result in detrimental transgenerational effects that decrease great-grand offspring fitness. We combined theoretical modeling and experimental approaches to investigate multigenerational fitness trade-offs induced by larval starvation in *Caenorhabditis elegans*. We demonstrate that larval starvation triggers a cascading effect: starved individuals suffered marked fitness losses, their direct offspring enjoyed fitness gains in both starvation and ad libitum environments, but great-grand offspring paid fitness costs. Demographic simulation models revealed that genotypes exploiting this short-term intergenerational advantage outcompete rival genotypes despite the deferred transgenerational debt. Our findings demonstrate that adaptive intergenerational gains can be intrinsically linked to maladaptive transgenerational outcomes, challenging the assumption that transgenerational effects are inherently beneficial and highlighting the role of multigenerational trade-offs in evolution.

## Introduction

Intergenerational and transgenerational effects occur when the environmental experiences of parents or more distant ancestors influence the fitness of subsequent generations, potentially shaping evolutionary trajectories and ecological dynamics (Baduel et al., 2024; Baugh & Day, 2020; Bonduriansky & Day, 2009; Burton & Greer, 2022; Burton & Metcalfe, 2014; Webster & Phillips, 2025). While intergenerational effects involve immediate parental–offspring interactions that typically enhance offspring fitness, transgenerational effects extend beyond direct offspring, influencing fitness in later descendants. By convention, intergenerational effects are present in the F_1_, while, depending on when the P0 generation are exposed, transgenerational effects occur in the F_2_ or F_3_ generation onwards (Tando & Matsui, 2023). These effects have been commonly viewed as adaptive mechanisms allowing organisms to adjust their descendants’ phenotypes to match anticipated environmental conditions. Several studies have described potentially adaptive intergenerational effects, such as decreased oxidative stress under parasite load in birds (De Coster et al., 2012), increased reproductive fitness during drought in plants (Mojzes et al., 2021), and defensive head morphs under predator stress in *Daphnia* (Agrawal et al., 1999). However, in contrast, empirical evidence for adaptive transgenerational effects remains limited (Charlesworth et al., 2017; Ivimey-Cook et al., 2021; Khatib et al., 2024; Sánchez-Tójar et al., 2020; Shahmohamadloo et al., 2025).

Theoretical models have described two adaptive evolutionary hypotheses of transgenerational effects. Anticipatory effects “pre-arm” offspring, conferring fitness benefits to offspring experiencing the same stressful environment as their parents (Kronholm, 2022), while heritable bet-hedging increases offspring trait variance and, therefore, population survivability, through trading an immediate decrease of arithmetic mean fitness for an increase in, long-term, geometric mean fitness (O’Dea et al., 2016). While theoretical models provide compelling explanations for the adaptive value of transgenerational effects, further empirical tests are needed to assess their generality and ecological significance in the face of putative evolutionary trade-offs (Harris et al., 2025).

Inter- and transgenerational effects can transiently alter life-history traits. For instance, changes in parental diet can alter offspring lifespan and reproduction in *Drosophila* (Camilleri et al., 2024) and survival in nematodes (Webster et al., 2018). Because an organism’s resources in nature may often be limited, investment into a life-history trait may result in trade-offs with other traits. Such resource allocation trade-offs are a core component of life-history theory and can accompany phenotypic responses to environmental changes (Stearns, 1989). The fitness consequences of such trade-offs have been explored both within parental generations (P_0_) (Chapman et al., 2024) and their immediate offspring (F_1_) (Burton et al., 2021). For instance, Burton and colleagues found that offspring from nematodes exposed to osmotic stress exhibit intergenerational adaptations to oxidative stress but suffer a decrease in their pathogen response, giving evidence for an intergenerational trade-off between two phenotypically plastic traits (Burton et al., 2021). While existing work on multigenerational effects has focused on trade-offs between parents and their immediate offspring, the fitness consequences of long-term trade-offs remain largely unexplored. Such “multigenerational trade-offs” could arise when an adaptive effect in one generation of descendants (e.g., F_1_) imposes a fitness cost in a later generation (e.g., F_3_) or vice versa. Despite their potential significance for understanding how populations react to stress in changing and fluctuating environments, direct empirical tests for such multigenerational trade-offs are scarce.

Here, we used experimental and theoretical approaches to investigate intergenerational and transgenerational effects following larval starvation-induced developmental arrest in the nematode *Caenorhabditis elegans* (Figure 1). Previous work has shown starvation during the first larval stage (L1) results in an intergenerational increase in offspring provisioning (Hibshman et al., 2016; Jordan et al., 2019) and a transgenerational increase in starvation survival (Jobson et al., 2015). By taking multiple fitness components (lifetime reproductive success [LRS], age-specific reproduction, and rate-sensitive fitness and survival) across multiple different environments (Table S1), we found that, independent of their environmental conditions, descendants of starved individuals exhibit increased intergenerational (F_1_) fitness but decreased transgenerational (F_3_) fitness. Evolutionary simulation models of boom-and-bust population dynamics showed that maladaptive transgenerational effects can evolve as a cost of beneficial intergenerational effects. Together, our findings challenge the assumption that transgenerational effects are inherently beneficial and advance our understanding of how environmental stress can alter population dynamics in changing environments.

**Figure 1.**
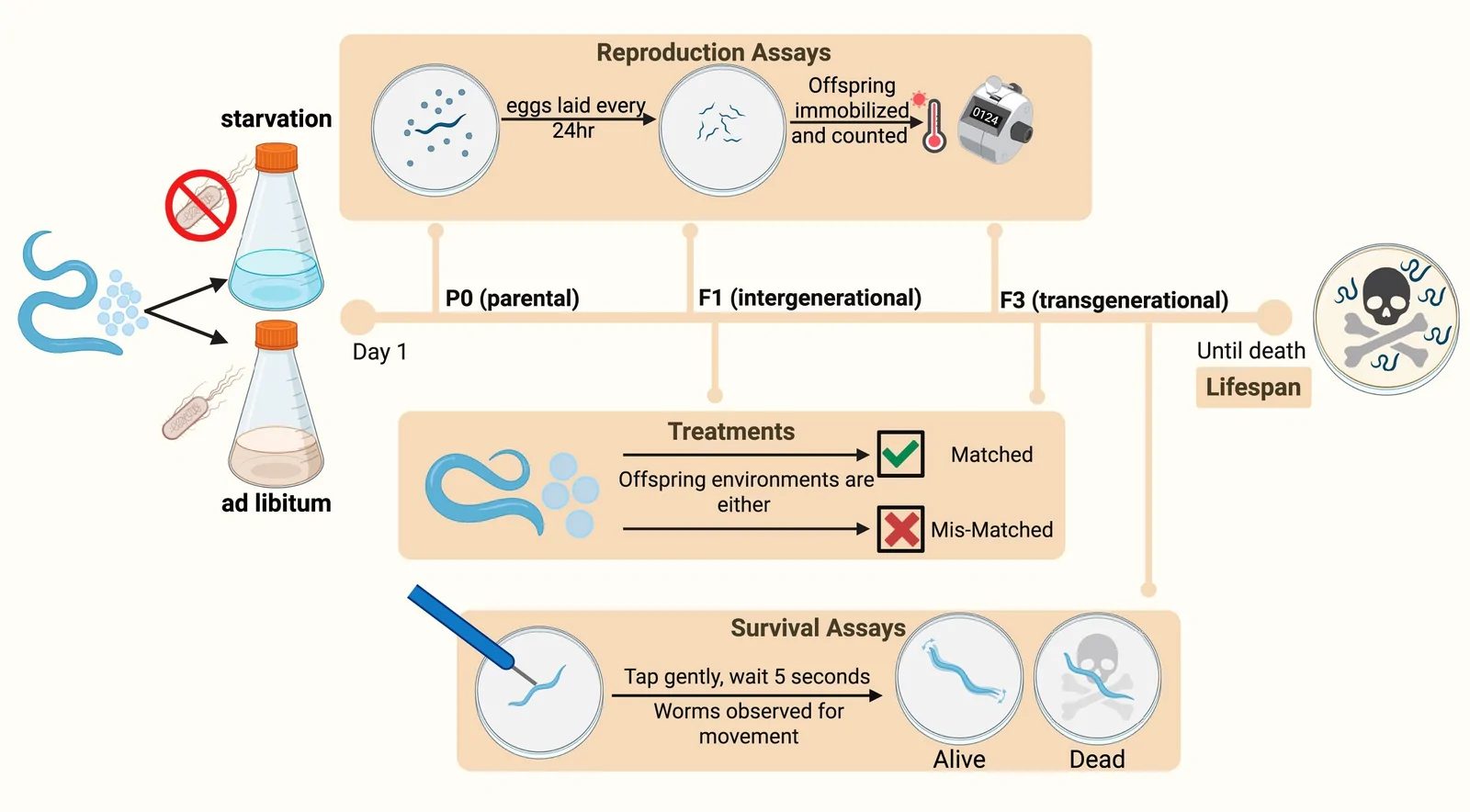
Experimental design. We placed fertilized *Caenorhabditis elegans* eggs into either an environment with no food or ad libitum food and allowed them to hatch. Worms that hatched in an environment with no food enter developmental arrest. We allowed worms to develop in ad libitum conditions after 7 days of developmental arrest or after 24 hr in control conditions. F1 and F3 offspring of P0 worms were either put back into the same or the opposite environment to the P0 worms (Table S1). F1 and F3 lines were run independently to each other and the F3 line was allowed to develop as normal through F1 and F2 generations. Fitness assays were taken in P0, F1, and F3 generations. For reproduction assays, worms were transferred to new plates every 24 hr, eggs were allowed to hatch and develop, and before the offspring reached reproductive age individuals were immobilized and each brood counted. Survival was assayed by gently touching worms each day and observing any movement. Worms unresponsive to touch were classed as dead. The figure was created with BioRender.com.

## Results

### P0

#### Age-specific reproduction

Reproductive timing was significantly affected by extended 7-day L1 larval starvation in the parental generation (ANOVA type III: *χ^2^ =* 155.98, *p <* 0.001), as indicated further by the spline interactions with age (Table S3). Individuals that experienced extended larval starvation had a delayed reproductive schedule compared to individuals raised in control conditions. Larval starvation resulted in a 1-day delay in both peak reproduction and onset of reproduction (Figure 2C), with most individuals starting reproduction on day 2 and peaking on day 3. Moreover, peak reproduction was decreased in larval starvation treatments.

**Figure 2.**
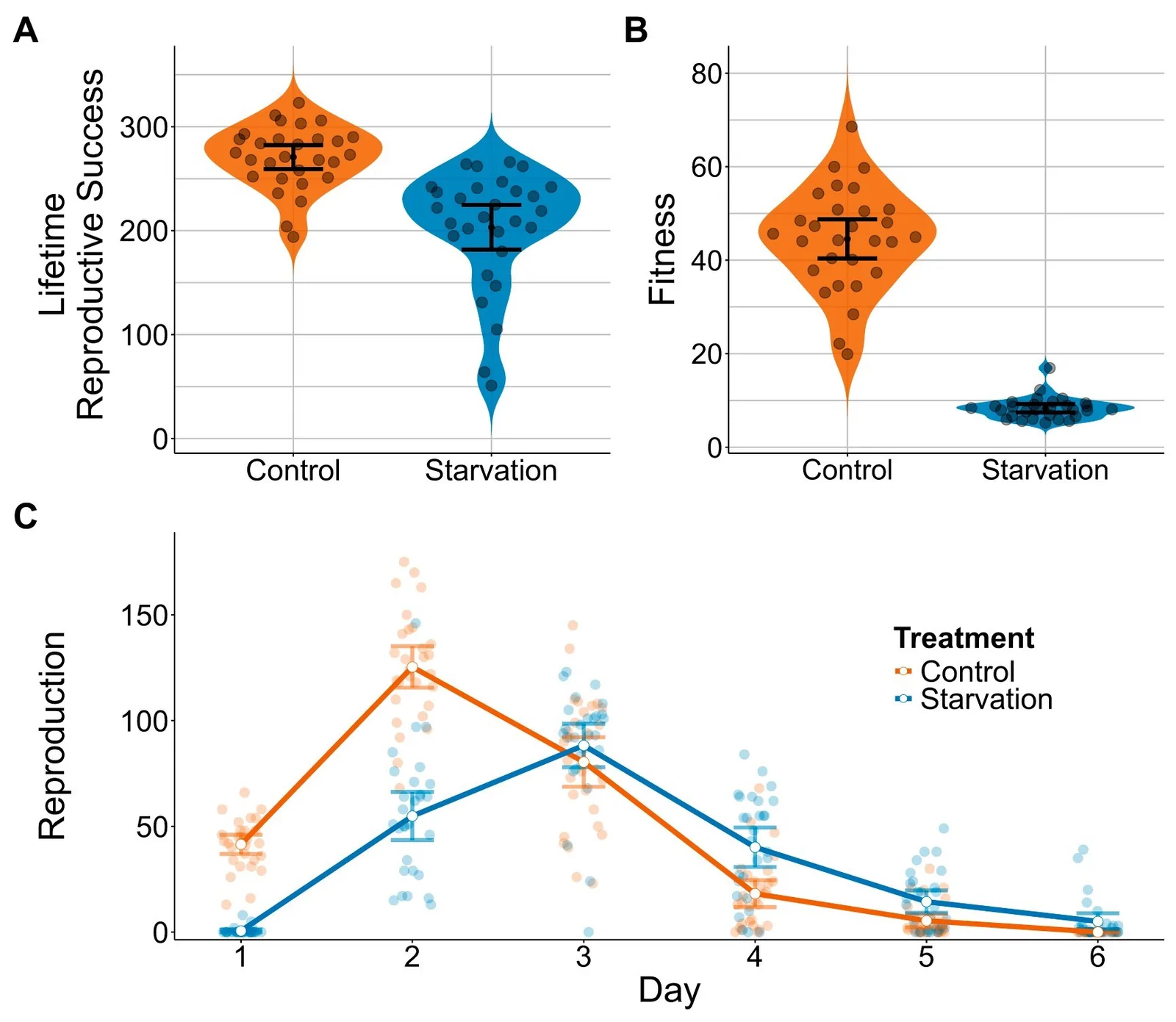
P0 fitness assays. P0 worms developed in either control (ad libitum) or larval starvation conditions. (A) The lifetime reproductive success, which is the total reproductive output per individual, (B) and rate sensitive fitness (λ), which represents the population growth potential. (C) Plotted is daily reproduction, with line connecting the mean values for each treatment on each day. Each point represents one individual. All error bars represent 95% CIs.

#### Fitness

Lifetime reproductive success was significantly decreased; individuals that experienced extended larval starvation had on average a 27% decrease in reproductive output (Estimated Marginal Mean (EMM) Wald *z-*test*: z = −*5.62, *p <* 0.001) (Figure 2A) (Table S6). A delay in reaching peak reproduction along with an overall decrease in total reproduction resulted in a lower rate-sensitive fitness (*λ*_ind_) (81% decrease in starved individuals), for individuals subjected to larval starvation (EMM Wald *z-*test: *z = −*24.62, *p <* 0.001) (Figure 2B; Table S9).

#### Survival

The survival of P_0_ individuals was significantly reduced by larval starvation (EMM Wald *z-*test: *z* = 2.21, *p =* 0.027) (Figure S1 and  Table S12). Individuals who underwent larval starvation exhibited significant increases in risk of mortality (hazard), in comparison to individuals who developed in ad libitum conditions (log odds *= −*0.61, 95%: −1.12, −0.092, *p =* 0.0207).

### Intergenerational and transgenerational effects

#### Age-specific reproduction

Reproductive timing was significantly influenced by treatment in the F_1_ generation (ANOVA type III: *χ*^2^*=* 178.79, *p =* <0.001) (Figure 3E). Interactions with age were significant for most splines (see the Supplementary Material for model outputs). Larval starvation in the F_1_ generation caused delayed peak reproduction regardless of parental treatment (Table S4) (**Offspring treatment (Parental treatment)**, EMM Wald *z-*test: **F_1_Control(P_0_Control) − F_1_Starvation(P_0_Control)**: *z*: −13, *p* < 0.001, **F_1_Control(P_0_Starvation) − F_1_Starvation(P_0_Starvation)**: *z*: −11.84, *p* < 0.001). However, F_1_ worms from starved parents reproduce more earlier than those from control parents regardless of their environment (EMM Wald *z*-test: **F_1_Control(P_0_Control) − F_1_Control(P_0_Starvation)**: *z* = 3.29, *p* = 0.006, **F_1_Starvation(P_0_Control) − F_1_Starvation(P_0_Starvation)**: *z* = 6.79, *p* < 0.001). Reproductive timing of F_3_ (great-grand offspring) was significantly affected by ancestral treatment interactions in the splines of age, altering the shape of their age-related reproduction curves (Figure 3F; Table S5) (ANOVA type III: *χ*^2^*=* 324.8, *p <* 0.001).

**Figure 3.**
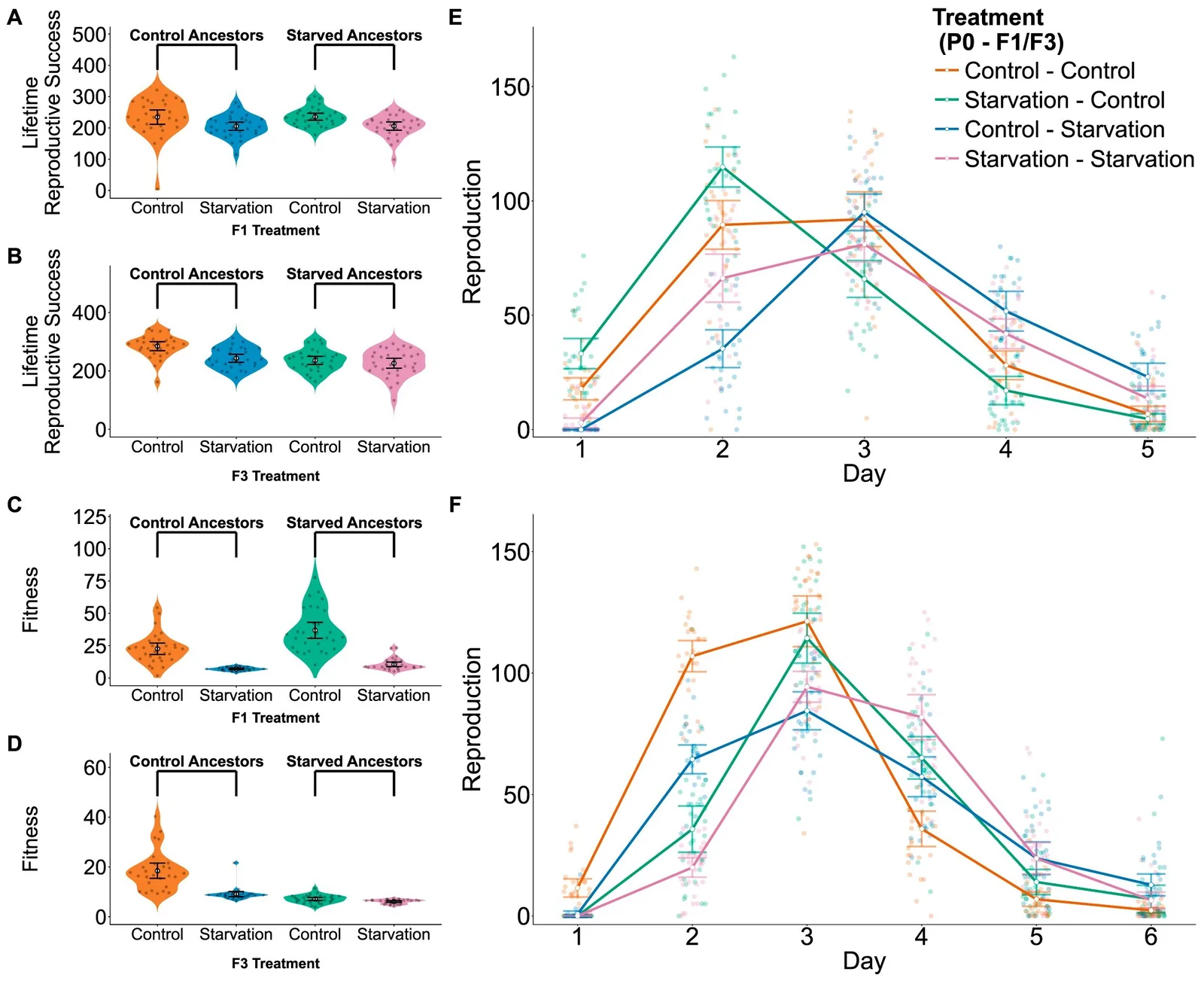
F1 and F3 fitness assays: violin plots show lifetime reproductive success (A and B) and rate-sensitive fitness (λ_ind_) (C and D) of individuals from different treatment combinations across generations. (E and F) Daily reproduction across days of adulthood. Treatments represent combinations of parental P0 and F1 (A, C, and E) or F3 (B, D, and F). Plotted is daily reproduction, with line connecting the mean values for each treatment on each day. Each point represents one individual. All error bars represent 95% CIs.

#### Fitness

F_1_ individuals reared in control conditions had significantly higher LRS than those reared in starvation conditions, irrespective of the parental treatment (EMM Wald *z*-test: **F_1_Control(P_0_Control) −F_1_Starvation(P_0_Control)***: z =* 3.46, *p* = 0.003; **F_1_Control(P_0_Control) −F_1_Starvation(P_0_Starvation)**: *z =* 3.09, *p* = 0.011; **F_1_Control(P_0_Control) −F_1_Starvation(P_0_Control)**: *z =* 3.49, *p* = 0.003; **F_1_Control(P_0_Starvation) −F_1_Starvation(P_0_Starvation)**: *z =* 3.02, *p =* 0.014). However, based on parental treatment, there was no difference in LRS in F_1_ individuals while in control conditions (EMM Wald *z-*test: **F_1_Control(P_0_Control) − F_1_Control(P_0_Starvation)**: *z =* 0.75, *p =* 0.88, Figure 3A). Similarly, there was no difference in LRS of individuals who underwent F_1_ larval starvation between parental treatments (EMM Wald *z*-test: **F_1_Starvation(P_0_Control) −F_1_Starvation(P_0_Starvation)**: *z =* −0.45, *p =* 0.97) (Figure 3C; Table S7).

Offspring of starved parents showed decreased LRS regardless of the environment they themselves experienced. Individuals’ LRS was significantly altered by their lineage (ANOVA type III: *χ*^2^*=* 35.06, *p* < 0.001) (Figure 3B). Specifically, individuals placed into control conditions from control great-grandparents had higher LRS than those who underwent F_3_ larval starvation (EMM Wald *z-*test: **F_3_Control(P_0_Control) − F_3_Starvation(P_0_Control)**: *z =* 3.71, *p =* 0.001, **F_3_Control(P_0_Control) − F_3_Starvation(P_0_Starvation)**: *z =* 5.45, *p <* 0.001). Furthermore, these individuals also had increased LRS compared to individuals placed in control environments from great-grandparents who underwent larval starvation (EMM Wald *z-*test: **F_3_Control(P_0_Control) − F_3_Control(P_0_Starvation)**, *z =* 4.49, *p <* 0.001). Conversely, the benefit to LRS when placed in an F_3_ control environment was lost in individuals from larval starvation lineages, with no differences in LRS when comparison to individuals raised in larval starvation conditions (EMM Wald *z-*test: **F_3_Control(P_0_Starvation) − F_3_Starvation(P_0_Control)**: *z =* −0.715, *p =* 0.89; **F_3_Control(P_0_Starvation) − F_3_Starvation(P_0_Starvation)**: *z =* 0.98, *p =* 0.76). Similarly, there was no effect of lineage on the LRS of F_3_ individuals placed in larval starvation in the F_3_ (EMM Wald *z-*test: **F_3_Starvation(P_0_Control) − F_3_Stavation(P_0_Starvation)**: *z =* 1.68, *p =* 0.33) (Table S8).

Rate-sensitive fitness was also significantly different between F_1_ lineages (ANOVA type III: *χ*^2^  *=* 291.58, *p <* 0.001) (Figure 3B; Table S10). Similarly to LRS, individuals in F_1_ control conditions had higher λ_ind_ than those who underwent F_1_ larval starvation regardless of parental treatment. For instance, worms from starved parents showed a 63% increase in starved and 49% increase in control environments of λ_ind_ when compared to control worms (EMM Wald *z-*test: **F_1_Control(P_0_Control) − F_1_Starvation(P_0_Control)**: *z =* 10.95, *p <* 0.001; **F_1_Control(P_0_Control) − F_1_Starvation(P_0_Starvation)**: *z =* 7.09, *p <* 0.001; **F_1_Control(P_0_Starvation) − F_1_Starvation(P_0_Control)**: *z =* 15.53, *p <* 0.001; **F_1_Control(P_0_Starvation) − F_1_Starvation(P_0_Starvation)**: *z =* 11.63, *p <* 0.001). However, a comparison of worms in the same F_1_ environment shows that offspring of starved parents had higher λ_ind_ than those from control parents (EMM Wald *z-*test: **F_1_Control(P_0_Control) − F_1_Control(P_0_Starvation)**: *z =* −4.58, *p <* 0.001, **F_1_Starvation(P_0_Control) − F_1_Starvation(F_1_Starvation)**: *z =* −3.76, *p <* 0.001).

In the F_3_ generation, ancestral treatment also had a significant impact on rate-sensitive fitness (ANOVA type III: *χ*^2^  *=* 406.23, *p* < 0.001) (Figure 3D; Table S11). Worms from a control great-grandparental lineage, placed in a control environment, had increased *λ*_ind_ when compared to individuals of any great-grandparental lineage that underwent larval starvation (EMM Wald *z-*test: **F_3_Control(P_0_Control) − F_3_Starvation(P_0_Control)**: *z* = 14.04, *p* < 0.001; **F_3_Control(P_0_Control) − F_3_Starvation(P_0_Starvation)**: *z* = 19.24, *p* < 0.001). Furthermore, regardless of environment, individuals from the control great-grandparental lineage had increased *λ*_ind_ compared to those from larval starvation lineages. Specifically, P0 larval starvation decreased rate-sensitive fitness by 59% in control and 29% in starved conditions when compared to worms from control ancestors (EMM Wald *z-*test: **F_3_Control(P_0_Control) − F_3_Control(P_0_Starvation)**: *z* = 16.72, *p* < 0.001; **F_3_Starvation(P_0_Control) − F_3_Starvation(P_0_Starvation)**: *z* = 7.66, *p* < 0.001).

### Survival

The parental diet of the F_1_ worms had a significant effect on offspring survival (ANOVA type III: *χ*^2^*=* 10.46, *p* = 0.001) (Figure S2C and  Table S13). However, the effect of offspring environment on survival was nonsignificant (ANOVA type III: *χ*^2^  *=* 2.65, *p* = 0.104). Overall, in the F_1_ there was no significant interaction between offspring and parental treatment for survival (ANOVA type III: *χ*^2^*=* 0.61, *p* = 0.44) (Figure S2A). Post hoc analysis suggests that offspring in the control environment had increased survival if descended from starved parents compared to those descended from control parents (**F_1_Control(P_0_Control) − F_1_Control(P_0_Starvation):** log odds *=* 0.729, 95%: 015, 1.31, *p* = 0.071). However, in starved environments offspring from starved parents did not differ significantly in survival in comparison to those from control parents (**F_1_Starvation(P_0_Starvation) − F_1_Starvation(P_0_Control)**: log odds *=* −0.5, 95%: −0.023, 1.01, *p =* 0.067).

The lineage of F_3_ worms had a nonsignificant effect on survival (ANOVA type III: *χ*^2^ = 3.43, *p* = 0.064) (Figure S2 and Table S14). The interaction between offspring and lineage treatment was significant for survival (*χ*^2^ = 5.09, *p* = 0.024). Post hoc analysis showed that F_3_ worms in a starvation environment, whose great-grandparents underwent larval starvation, displayed no significant change in survival, compared to those descended from control great-grandparents (**F_3_Starvation(P_0_Control) − F_3_Starvation(P_0_Starvation)**: log odds = 0.304, 95%: −0.29, 0.89, *p =* 0.55). In contrast, worms from well-fed great-grandparental lineages had decreased survival in starved conditions (**F_3_Control(P_0_Control) − F_3_Starvation(P_0_Control):** log odds = −1.12, 95%: −1.74, −0.512, *p* < 0.001) (Figure S2D).

### Comparing intergenerational and transgenerational effects

To compare the fitness effects of intergenerational and transgenerational effects, we ran models to test the interaction between offspring treatment, parental treatment, and generation. Specifically, we were interested in how LRS and rate-sensitive fitness changed in the intergenerational and transgenerational generation. We found that LRS did not differ between intergenerational and transgenerational starved individuals (EMM Wald *z-*test: *z = −*1.19, *p* = 0.24, *SE* = 0.084) (Table S15). However, rate-sensitive fitness was significantly lower in the transgenerational generation compared to the intergenerational generation (*z* = −5.20, *p* < 0.001, *SE* = 0.13) (Table S16), suggesting that differences between intergenerational and transgenerational effects are primarily driven by reproductive schedule rather than total fecundity.

### Simulations of transgenerational trade-offs

To explore the evolutionary dynamics of multigenerational trade-offs, we simulated boom-and-bust population dynamics of a natural population of nematodes (Félix & Duveau, 2012). Our simulations contained two strategies, one employing multigenerational trade-offs and one without. After 100 days. both strategies persisted in the population with their frequencies oscillating (Figure 4A). However, individuals with multigenerational trade-offs consistently represented a higher average proportion of the population. After 1,000 days, this advantage became more pronounced, with the multigenerational trade-off strategy almost reaching fixation (Figure 4B). A parameter search for different values of intergenerational and transgenerational relative fitness shows that multigenerational trade-offs can evolve when the long-term fitness benefits of intergenerational effects outweigh the short-term fitness costs of transgenerational effects (Figure S3). These results show that multigenerational trade-offs can confer long-term adaptive benefits in transient boom-and-bust populations.

**Figure 4.**
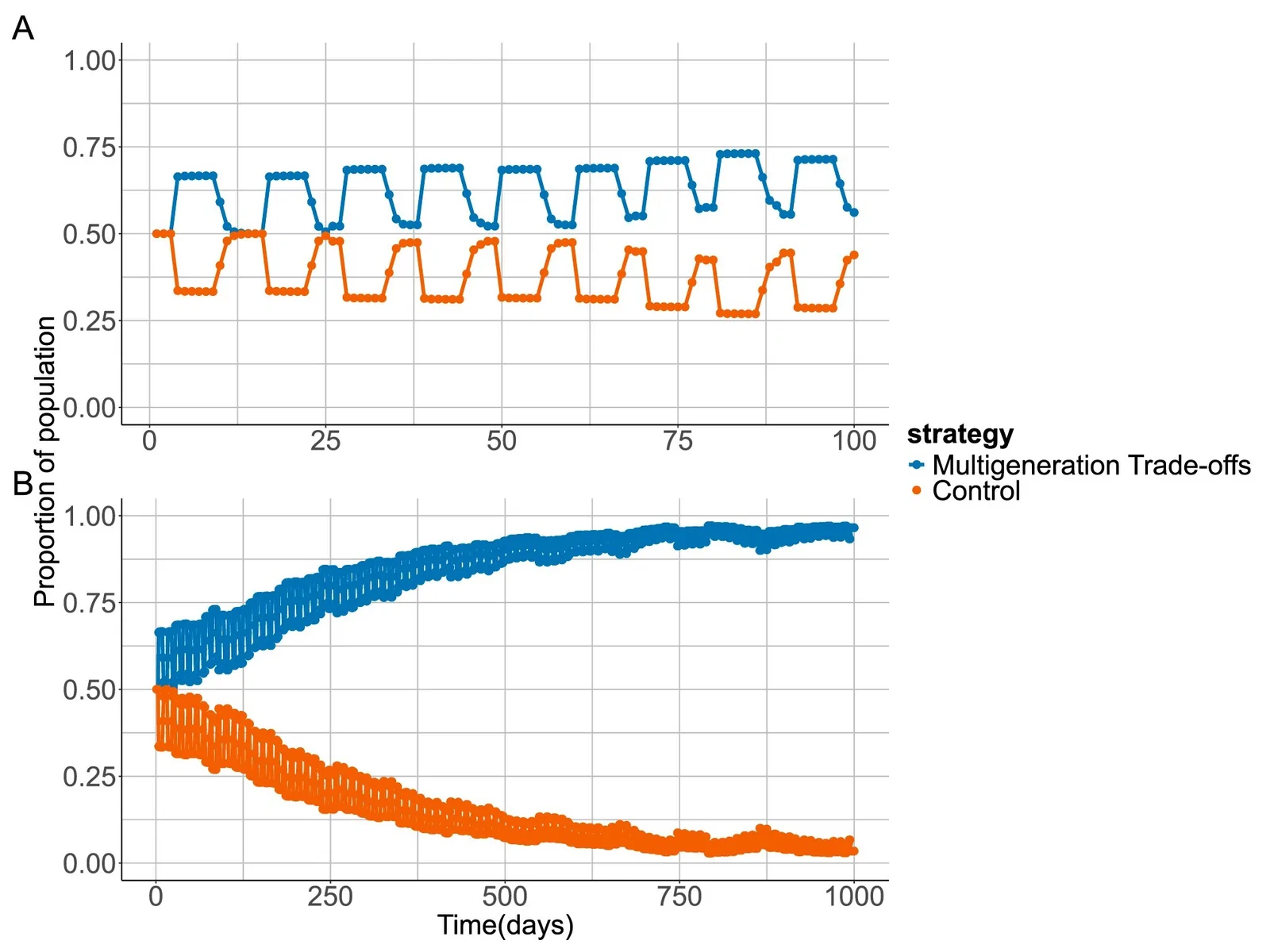
Evolutionary simulations of multigenerational trade-offs. Plots show the output of evolutionary simulations of boom-and-bust populations after 100 days (A) and 1,000 days (B). The simulations competed populations which utilized multigenerational trade-offs and those that did not.

## Discussion

Our results demonstrate that prolonged L1 arrest induced by starvation in *C. elegans* can induce adaptive intergenerational and maladaptive transgenerational effects. Across four complementary fitness estimates, lifetime reproductive output, age–specific fecundity, rate–sensitive fitness (λ), and survival, we detected these effects in both matched and mismatched environments, showing they are robust to the offspring’s rearing conditions. Our simulation model of boom-and-bust population dynamics further supports an evolutionary hypothesis, where short–term gains in the F_1_ generation are favored even though they carry costs in the F_3_ generation, because of the net benefit to these alleles. In other words, selection on adaptive intergenerational plasticity can inadvertently cause maladaptive transgenerational outcomes that are tolerated because of the overall fitness benefit. While previous studies have found beneficial transgenerational effects (Yin et al., 2019), our results indicate that transgenerational effects need not be adaptive and underscore the importance of estimating fitness trade-offs across multiple generations and environments to fully resolve how organisms react to fluctuating environmental conditions. Importantly, we show that maladaptive transgenerational effects can evolve as a cost of selection on beneficial intergenerational effects.

Consistent with previous research, we show that starvation can induce adaptive intergenerational effects in *C. elegans*. Early-life starvation has been shown to cause reproductive defects in the parental generation (Jordan et al., 2019). However, offspring from starved worms are more resistant to starvation and less susceptible to developmental germ line abnormalities (Harvey & Orbidans, 2011). Importantly, F_1_ offspring of starved individuals reproduce more and earlier, regardless of environment, which, in a growing population, confers an evolutionary advantage (Brommer et al., 2002). Our data suggest that the same environmental cue (here, starvation) that benefits F_1_ offspring may impose latent fitness costs two generations later. This finding cautions against assuming that intergenerational effects are uniformly beneficial even if they increase fitness in F_1_ generation. Instead, they can conceal delayed costs that only become visible when fitness is tracked beyond the first generation. Detecting such hidden costs will require the kind of multigeneration, multienvironment assays we deployed here. In this study, intergenerational benefits outweighed transgenerational costs, but this result may not be uniform across all taxa and environments. Furthermore, additional trade-offs could be present in morphological traits, driving differences in competitive ability. To evaluate the generality and morphological cause of multigenerational trade-offs, we suggest that future work should utilize multiple evolutionary replicates and morphometric data.

Transgenerational effects have been well documented, and *C. elegans* provide a powerful tool for empirical tests of evolutionary hypotheses related to these effects. For instance, great-grand offspring from starved worms have been shown to have increased variability in stress-related phenotypes, which was hypothesized to be a form of diversifying bet-hedging (Jobson et al., 2015; Starrfelt & Kokko, 2012). In contrast, we observed a marked reduction in variance for rate-sensitive fitness, in starvation lineage groups (Figure 3C and D), suggesting an alternate evolutionary strategy. For instance, our results align with canalization through transgenerational developmental plasticity, as F3 worms from starved ancestors display constrained phenotypic variation compared to controls (i.e., reproductive timing and rate-sensitive fitness). Previous work has shown great-grand offspring from worms that have undergone an extended dauer life stage (an alternate dispersal life stage) have increased starvation resistance, suggesting transgenerational anticipatory effects (Webster et al., 2018). Theoretical work has shown that both bet-hedging and anticipatory effects are likely to evolve depending on the predictability of future environments (Draghi, 2023; Kronholm, 2022), and in some cases both strategies can be present simultaneously (Joschinski & Bonte, 2020). Both bet-hedging and anticipatory effects emphasize adaptive outcomes across generations. However, we show here that transgenerational effects can be maladaptive. Furthermore, our simulations suggest maladaptive transgenerational effects can be selected for if there are intergenerational benefits. The results outline a distinct evolutionary dynamic, that maladaptive transgenerational effects can emerge as a trade-off with intergenerational benefits. This contrasts with prior evolutionary models of transgenerational inheritance and provides an explanation for the existence of maladaptive transgenerational effects.

While experimental and theoretical work has identified adaptive intra- (Chapman et al., 2024; Travers et al., 2021) and intergenerational trade-offs (Bliard et al., 2024; Mignerot et al., 2023), longer-term trade-offs have rarely been considered or documented. Our model shows that maladaptive transgenerational effects can evolve as a byproduct of selection on beneficial intergenerational effects when the transgenerational cost is mitigated by ecological dynamics. In *C. elegans*, for example, populations grow exponentially until they reach a local carrying capacity, at which point developing individuals enter an alternative dispersal life stage (Frézal & Félix, 2015). Such boom-and-bust population dynamics are common in natural populations of nematodes and may mitigate the cost of maladaptive transgenerational effects facilitating their evolution, when there is a periodical resetting of environmental conditions. Our simulation model suggests that the evolution of multigenerational trade-offs depends on the magnitude of fitness effects, generation time, and periodical environment resetting. Beyond nematodes similar conditions, which meet the criteria for the evolution of multigenerational trade-offs, have been documented in zooplankton, annual plants, and certain insect species (Gremer & Venable, 2014; Rother et al., 2010; Schebeck et al., 2024). However, in a stable environment, transgenerational effects may have longer-term fitness consequences, leading to different evolutionary outcomes (Figure 5). We acknowledge that our models are parameterized on nematode data and may not translate directly to other similar taxa; the magnitude and/or direction of effects could vary significantly between study system. As such, testing for context–dependent outcomes in taxa with similar and contrasting population dynamics will be essential for determining how general multigenerational trade–offs are and which ecological settings favor their evolution.

**Figure 5.**
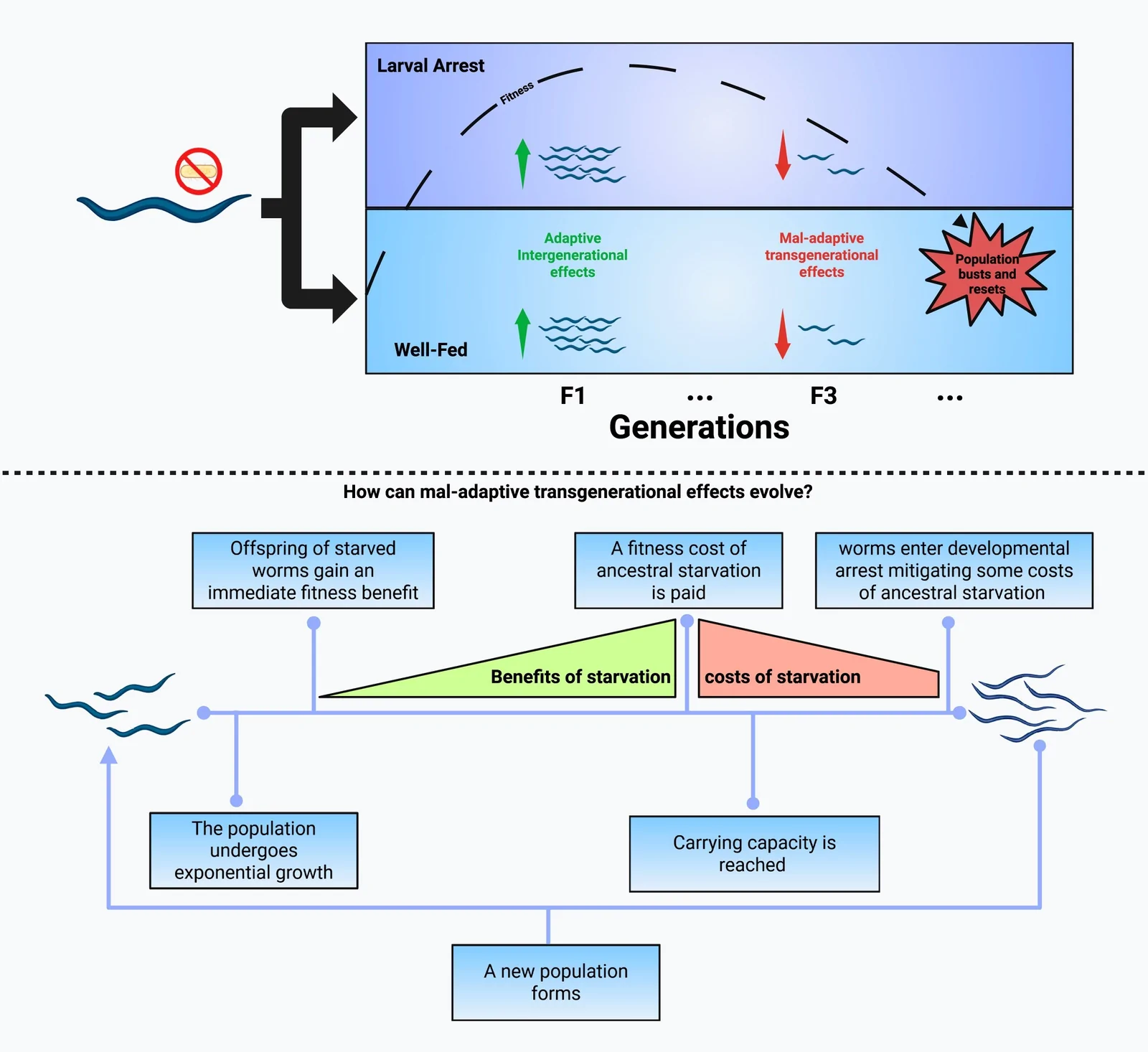
How can maladaptive transgenerational effects evolve? Illustration showing how larval starvation can generate both adaptive intergenerational and maladaptive transgenerational fitness effects. When larvae experience starvation and enter developmental arrest, their immediate offspring may exhibit adaptive intergenerational benefits. However, the cost of immediate beneficial effects is paid in a later generation. Worms enter developmental arrest due to the population reaching the carrying capacity, buffering some costs of ancestral starvation. Overall offspring receive more from the immediate benefits of larval starvation than future generations pay in eventual costs. The figure was created with BioRender.com.

We did not directly investigate the molecular mechanisms of transgenerational effects, but previous work has shown that larval starvation can induce the production of small RNAs that are inherited by great-grand offspring in *C. elegans* (Rechavi et al., 2014). However, it is unlikely that multigenerational trade-offs are mediated through small RNAs alone. However, alternate possibilities exist; for instance, intergenerational effects could arise from increased maternal provisioning, and transgenerational effects due to a trade-off between transposable element (TE) activity and gene expression (Hollister & Gaut, 2009). Previous work has shown that stressful environments can alter TE activity (Capy et al., 2000). Notably, the silencing of TEs can have bystander effects such that genes adjacent to silenced TEs show altered expression and can produce maladaptive phenotypes in later generations (Hollister & Gaut, 2009; Miller et al., 2023). The “TE-mediated trade-off” model provides a framework to address unanswered questions about transgenerational effects. For instance, while this model still accommodates previous observations of sRNA inheritance, it can also explain maladaptive effects as a cost of silencing selfish genetic elements. However, although this model has some theoretical support (Huang & Lee, 2024), future work is needed targeting small RNA pathways and TEs to determine the mechanisms that underlie long-term fitness costs of parental stress and shape multigenerational trade-offs.

Together, our results show that extended larval starvation in *C. elegans* results in beneficial intergenerational effects and detrimental transgenerational effects, regardless of environmental conditions experienced by the offspring. Eco–evolutionary simulation models show that such maladaptive F3 outcomes can arise as a byproduct of selection for adaptive intergenerational plasticity, overturning the common assumption that transgenerational effects are inherently beneficial. These findings underscore the need to measure fitness across multiple generations and ecological contexts to fully understand adaptive plasticity. Little is known about multigenerational trade–offs in other taxa and closing this gap will require experiments that utilize multigenerational fitness assays in organisms with diverse life histories across the tree of life. Integrating multigenerational trade-offs with ecological and evolutionary perspectives will help us predict how populations navigate fluctuating environments across generations.

## Methods

### Strains and maintenance

*Caenorhabditis elegans* nematodes of the N2 Bristol strain were used in all assays. All nematodes were ordered from the *Caenorhabditis* Genetics Centre, which is funded by NIH Office of Research Infrastructure Programs (P40 OD010440). Populations were initiated from thawed N2 worms from −80 °C and maintained for at least three generations before bleaching. When not undergoing treatments and during fitness assays, nematodes were maintained on NGM (Nematode Growth Medium) agar plates seeded with a lawn of *Escherichia coli* OP50.1P (90 mm for maintenance and 35 mm for assays) and kept in climate chambers set to 20 °C, 60% relative humidity, and constant darkness. NGM agar contained antibiotics (100 µg ml-1 ampicillin) and a fungicide (100 µg ml-1 nystatin). See Wormbook (http://www.wormbook.org/) for details on OP50 cultures, S-buffer, and M9 recipes.

### Intergenerational and transgenerational fitness assays

Our experimental design resulted in 10 different treatments, two parental treatments consisting of either a 7-day larval starvation or control conditions (fully fed); F1 and F3 offspring had either a matched or mismatched diet to their parents or great-grandparents, respectively. To control for different developmental times across treatments, worms are plated for reproduction regardless of life stage. Under control conditions, worms typically took 36–48 hr to reach late L4 after treatments, Therefore, this range was used to set up reproduction. Fitness assays were set up by randomly picking individual late L4 worms onto individual 35-mm NGM seeded plates. For the next 6–7 days every 24-hr worms were transferred to new plates. Any eggs laid on the plates were allowed to develop for 2 days before being heat-shocked at 42 °C for ∼3 hr and then counted. Worms were censored if they went missing, and worms that died before the end of reproduction (e.g., due to matricide) were scored as having 0 reproduction from that point.

Survival assays were conducted on 35-mm seeded OP50.1P NGM plates. Ten worms were plated per plate, and worms were transferred every 2 days, or 1 day during reproduction. Lifespan was checked every day; death was defined as the absence of worm movement after a light touch. A worm was censored if it died due to unnatural circumstances, such as matricides or going missing.

### Statistical analysis and simulations

All statistical analysis and simulations were conducted using R version 4.3.2 (Posit Team, 2025). For the P_0_ models, a fixed effect of treatment was fitted along with a random effect of “plate” to account for potential pseudo-replication. For F_1_ and F_3_ models, treatment consisted of the worm’s lineage resulting in a factor of 4 levels, “plate” was fitted as a random variable.

We analyzed four fitness estimates using generalized linear mixed effect models. The first of which, age specific reproduction, required further fixed effects of “Day” along with a spline of “Day” and its interaction with “Treatment.” Age-specific reproduction often shows significant dispersion and zero-inflation in *C. elegans*. Therefore, model diagnostics were run using the DHARMa package, to check for this (Hartig et al., 2024). Overdispersion and zero-inflation were always addressed. Underdispersion in F1 and F3 data could not be solved; however, since it usually makes results more conservative, we decided to ignore it. Multiple error distributions were fitted using the GlmmTMB package (see the Supplementary Material) and age-specific reproduction curves were visualized using GGplot2 (Brooks et al., 2025; Wickham et al., 2025).

The second estimate of fitness we analyzed was LRS (total number of offspring produced per individual). Here, a basic generalized linear mixed model consisting of either a negative binomial or Poisson error structure and fixed effects of treatment, lineage (which treatment their ancestor underwent), and a random effect of “Plate” was used. We also fitted an alternative estimate of fitness, rate-sensitive fitness (*λ*_ind_), which can be estimated through extracting the dominant eigenvalue of individual structured Leslie matrices of life-history data (Brommer et al., 2004). *λ*_ind_ was fitted using a gamma error structure and the same fixed and mixed effects as LRS. Reproductive fitness estimates were visualized using GGplot2.

Our final fitness estimate was survival. Only natural deaths were included in the analysis; matricides and lost worms were censored. Most data conformed to the assumptions of a Cox proportional hazard model, except data from the F_1_ worms. Therefore, we used an event history analysis for all data for consistency. Since the model requires sampling from every day, we added a random effect of “Worm ID” to account for the repeated measures. The output from the model was then visualized through a forest plot and survival curve using ggplot2.

To test if there was a difference between fitness in the intergenerational and transgenerational generation, two extended models were fitted for LRS and rate-sensitive fitness. These models included a three-way interaction between parental environment, offspring environment, and generation. The error distribution and random effects were fitted as previously described.

In all cases, model selection was performed to identify the best zero-inflation and dispersion parameters for all response variables (see the Supplementary Material for model information). Akaike information criterion (AICs) were then compared before the final model assumptions were checked using the DHARMa package. Finally, to determine the overall effect of lineage in the F_1_ and F_3_, pairwise tests were conducted using the emmeans package, which estimates marginal means (Lenth et al., 2025). If the model included an interaction term, then the emtrends function was used instead, which compares the estimated marginal slopes. Outputs from these functions were run through the pairs function for pairwise analyses.

Simulations were run using on RStudio (Posit team, 2025), based on collected data and grounded in existing knowledge of nematode ecology. The simulations explored the effects of competing two transgenerational strategies within a boom-and-bust nematode population. The first strategy simulated multigenerational trade-offs, which after starvation displayed F_1_ increases and F_3_ decreases in fitness with an intermediate point at F_2_. The other strategy exhibited no transgenerational effects, i.e., constant fitness. We assumed each strategy reacted the same way in the exposed (parental) generation, and the difference was in their intergeneration and transgenerational effects. The simulation input consists of “cohorts” of worms containing information on the worms age, brood size, generations since starvation, and transgenerational strategy. Global parameters determined the number of bacterial cells (food), age-specific reproduction/feeding, and the relative fitness consequences of transgenerational effects (Table S2). The simulation was run through iterations, each representing 1 day in which the worms eat food, age, lay eggs, and with new broods being added to the population size as a cohort, sharing parental transgenerational strategy, with the process being repeated until a set number of days was reached. When the population runs out of food a starvation event is triggered, and the worms enter the dispersal life stage. Worms in larval stages “disperse” to find a new food source; the simulation now follows a new population formed of dispersed larvae. Simulation outputs were visualized using ggplot2.

## Supplementary Material

rag031_Supplemental_File

## Data Availability

R code and data used in all analysis and simulations are deposited at https://github.com/Isaac2181/Multigenerational-Trade-offs
